# SOCIAL WORKERS’ AND NURSES’ VIEWS ON COLLABORATIVE WORK IN A SETTING WITH SKILLED NURSING CARE

**DOI:** 10.1093/geroni/igad104.2049

**Published:** 2023-12-21

**Authors:** Tina Kilaberia, Dawn Apgar, Deborah Padgett, Bei Wu, Teri Kennedy

**Affiliations:** New York University, New York City, New York, United States; Seton Hall University, South Orange, New Jersey, United States; New York University, New York City, New York, United States; New York University, New York City, New York, United States; University of Kansas Medical Center, Kansas City, Kansas, United States

## Abstract

This study compares social workers’ and nurses’ views on collaborative work using descriptive and thematic analysis of semi-structured in-depth interviews conducted at an urban retirement and assisted living community including independent living, assisted living, and skilled nursing care in a United States Midwestern city. Social workers (N=11) and nurses (N=12) responded to questions about how they spent their time, with whom (job category) they worked most and least closely, and what constrained collaborative work. Descriptive analysis showed that social workers exercised greater collaborative awareness than nurses and viewed nurses as partners with whom they worked most closely, whereas nurses did not view social workers and non-clinical peers as such. Qualitative analysis showed that social workers were more focused on resident self-determination, advocacy, and requisite care whereas nurses’ orientation was directed toward safety, tasks, and clinical outcomes. In terms of collaborative work, the devalued professional identity of social workers was a threat to collaborative work whereas nurses portrayed failures in collaborative work as stemming from individual actions and attributes. Different approaches to collaborative work between social workers and nurses can impede teamwork. Interprofessional training focused on long-term services and supports may help to reconcile disparate approaches and strengthen collaborative work between social workers and nurses.

